# Predicting task performance from biomarkers of mental fatigue in global brain activity

**DOI:** 10.1088/1741-2552/abc529

**Published:** 2021-03-08

**Authors:** Lin Yao, Jonathan L Baker, Nicholas D Schiff, Keith P Purpura, Mahsa Shoaran

**Affiliations:** 1 Frontiers Science Center for Brain&Brain-machine Integration, Zhejiang University, Hangzhou, Zhejiang 310000, People’s Republic of China; 2 College of Computer Science, Zhejiang University, Hangzhou, Zhejiang 310000, People’s Republic of China; 3 School of Electrical and Computer Engineering, Cornell University, Ithaca, NY 14850, United States of America; 4 Feil Family Brain and Mind Research Institute, Weill Cornell Medicine, New York, NY 10021, United States of America; 5 Institute of Electrical Engineering and Center for Neuroprosthetics, Swiss Federal Institute of Technology (EPFL), Geneva 1202, Switzerland

**Keywords:** vigilance task, ECoG, mental fatigue, machine learning, non-human primates, traumatic brain injury

## Abstract

**Objective.:**

Detection and early prediction of mental fatigue (i.e. shifts in vigilance), could be used to adapt neuromodulation strategies to effectively treat patients suffering from brain injury and other indications with prominent chronic mental fatigue.

**Approach.:**

In this study, we analyzed electrocorticography (ECoG) signals chronically recorded from two healthy non-human primates (NHP) as they performed a sustained attention task over extended periods of time. We employed a set of spectrotemporal and connectivity biomarkers of the ECoG signals to identify periods of mental fatigue and a gradient boosting classifier to predict performance, up to several seconds prior to the behavioral response.

**Main results.:**

Wavelet entropy and the instantaneous amplitude and frequency were among the best single features across sessions in both NHPs. The classification performance using higher order spectral-temporal (HOST) features was significantly higher than that of conventional spectral power features in both NHPs. Across the 99 sessions analyzed, average F1 scores of 77.5%±8.2% and 91.2%±3.6%, and accuracy of 79.5%±8.9% and 87.6%±3.9 % for the classifier were obtained for each animal, respectively.

**Significance.:**

Our results here demonstrate the feasibility of predicting performance and detecting periods of mental fatigue by analyzing ECoG signals, and that this general approach, in principle, could be used for closed-loop control of neuromodulation strategies.

## 1. Introduction

Performance of simple tasks, when repeated over extended periods of time, requires considerable ‘mental effort’ [1]. As the number of repetitions increases along with time on task, error rates increase and subjects report experiencing a sense of ‘mental fatigue’. It is well known that the ability to be vigilant, to maintain attention on specific aspects of a task over time, degrades as arousal level and motivation decrease. These changes in attentiveness and engagement correlate with changes in physiology: reaction times slow, blood flow to the anterior forebrain decreases[2], and wide-spread synchronization of brain activity in the theta and alpha bands increases [3].

While most healthy subjects will experience a decrease in vigilance while performing tasks requiring focused attention over extended periods, individuals who have suffered a traumatic brain injury (TBI) are more quickly fatigued by the repetition of even simple tasks [4]. Furthermore, during periods of ‘mental fatigue’ these individuals demonstrate physiological responses similar to normal subjects executing difficult tasks over long periods of time [5]. Currently, no medication or device-based therapies can treat chronic dysfunctions of arousal regulation and cognition in these patients.

We previously demonstrated [6] that therapeutic use of central thalamic deep brain stimulation (CT-DBS) was able to restore fluent communication, executive function and motor control in a severe TBI patient who had been in a chronic minimally conscious state (MCS) for six years prior to CT-DBS. In a more recent study, an individual who for 18 years suffered from chronic mental fatigue and impaired cognitive function following a severe TBI, showed a concomitant restoration of executive function and a marked reduction of mental fatigue and daily napping with the application of CT-DBS [7]. Thus, DBS, when delivered during periods of mental fatigue, could potentially alleviate periods of inattention, drowsiness, and confusion experienced by the majority of TBI patients. While most therapeutic applications of neurostimulation are open loop, that is without feedback control linked to neuronal activity or the patient’s state, the greatest promise for these patients may depend on the development of closed-loop DBS technologies. However, the key to an effective closed-loop system will be the ability to robustly determine the onset or persistence of mental fatigue by analyzing brain activity in real-time. This general approach was successful in closed-loop (or adaptive) stimulation strategies for epilepsy [8, 9], movement disorders such as Parkinson’s [10, 11], and memory recall in epilepsy patients [12].

Effective closed-loop control could be implemented by identifying robust electrophysiological biomarkers linked to shifts in arousal and cognitive state. In prior studies we showed that behavioral performance in a sustained attention task correlates with marked changes in neural activity in the central thalamus [13], prefrontal cortex and dorsal striatum of intact NHPs [14]. For example, during the delay periods of correctly performed trials, firing rates of single neurons in the central thalamus significantly increased [13], and the power spectra of local field potentials (LFP) recorded in the frontal cortex and striatum, markedly shifted from lower to higher frequencies [14]. These electrophysiological signatures were linked to behavioral performance and state and were robust across experimental sessions and animals and inspired our approach here to seek additional features based on the spectral and temporal characteristics of global cortical activity that could be used in closed-loop control of DBS.

A key component of an effective closed-loop control system is a robust and efficient method for identifying electrophysiological biomarkers linked to specific brain states. The development of signal processing tools for the automated detection of shifts in arousal, task engagement, wakefulness, and vigilance has received considerable attention in human studies [15]. Readily recognizable features of the electroencephalogram (EEG), such as spectral peaks in the theta, alpha, and beta bands, which have been demonstrated to provide a window into brain states of arousal and consciousness [16], have led to the development of signal processing tools for automatic detection of brain states. The buildup of sleep and drowsiness has been characterized by increases in the theta and alpha activities as well as a decrease in the beta band [17, 18]. Studies employing EEG in humans, as they perform simple computer-based tasks [19–23] and neuropsychological tests [24], demonstrated consistent shifts in frontal theta and midline alpha power in the EEG. A combination of EEG sub-bands such as [theta + alpha]/beta was also shown to be useful for the detection and quantification of alertness levels [25].

However, more advanced decoders based in part on machine learning (ML) that employed multiple spectral power features from each EEG channel, showed significant improvement over conventional methods for predicting a driver’s vigilance in real time [26]. For example, support vector machine (SVM) classifiers can be trained on spectral features recorded from the occipital cortex to detect sleep or states of vigilance at a level of performance *>*90% [26–28]. In a study of auditory vigilance, where subjects were required to perform an auditory vigilance task every hour over a 25 h period of sleep deprivation, a probabilistic multi-class SVM utilizing the activity in the delta, theta, alpha, and beta bands in the EEG, had an accuracy of 87.2% in assigning the subjects to a behaviorally scored level of mental-fatigue based on the EEG signals alone. In another study [20], a mental arithmetic task was continuously performed by the subjects until they quit from exhaustion or 3 h had elapsed. It was shown that the induced mental fatigue was associated with increased power in parietal alpha and frontal theta. By employing the spectral features of EEG and a kernel partial least squares classifier, the classification accuracy between alert and fatigued task periods reached 91 %–100%.

The majority of previous efforts to detect mental fatigue and drowsiness have focused on classical spectral power features [29]. However, in a recent study, a complex spectrum-based decoder was proposed for the prediction of eye movement goals from LFPs in two macaque monkeys during memory-guided saccade tasks [30], demonstrating a significant performance improvement over conventional spectrum-based decoders. Moreover, spectral power features, in contrast to a network measure such as fixed canonical correlation, were not able to distinguish task/non-task engagement in humans [31].

Here we trained two NHPs to perform a visuomotor reaction-time task similar to those used to study vigilance and performance in humans [32–36]. In a prior study [14] we analyzed neuronal activity recorded from the prefrontal cortex and dorsal striatum to study the mechanisms of CT-DBS during task performance. In this study, we analyze the electrocorticography (ECoG) activity that was recorded broadly across all cortical regions in the same NHPs [14] as they performed the task across experimental sessions. We used simple spectral features in addition to higher order spectral-temporal (HOST) features such as wavelet entropy, instantaneous amplitude and frequency, measures of inter-areal neural interaction or connectivity (e.g. partial directed coherence and phase locking index), to develop a classifier for predicting when the animals failed to perform the task correctly. Extension of this general approach to real-time applications and ultimately for closed-loop stimulation is the subject of our ongoing and future studies.

## 2. Data acquisition and methods

The goal of this study was to classify performance using modern machine learning (ML) techniques combined with spectrotemporal and connectivity (coherence) biomarkers extracted from multi-channel ECoG signals recorded widely across cortical regions of healthy behaving non-human primates (NHP). The results described here are a first step in the development of an algorithm that can predict performance decrements associated with mental fatigue in healthy animals. Such algorithms could be integrated into adaptive (i.e. closed-loop) deep brain stimulation strategies to restore performance when periods of mental fatigue are predicted.

### 2.1. Electrodes and non-human primates

The use of non-human primates (NHP) as a model system in neuroscience [37] and in the development of DBS for movement disorders [38] is well-established. The frontal cortex of NHPs is structurally and functionally similar to humans [39, 40] and their ability to learn and perform a wide variety of complex cognitive tasks [41] makes them an ideal model to study mechanisms of arousal regulation during cognitively demanding tasks over extended periods of time [42].

All experiments were performed in strict accordance with the National Institutes of Health guidelines for use of animals in research and under an approved protocol from the Weill Cornell Medical College Institutional Animal Care and Use Committee (IACUC). A detailed description of the surgical techniques, behavioral control and data acquisition systems can be found elsewhere [43, 14]. In brief, two male adult monkeys (10 and 11 kg), macaca mulatta, were trained over the course of 6–8 months to sit quietly in a special-purpose primate chair (Crist Instruments Company, Hagerstown, MD) and carry out behavioral tasks in order to receive sips of water. With positive reinforcement alone, it was possible to train the animals to move calmly from their home cage to the chair, accept head restraint, work for water on a wide range of cognitive tasks, and to return calmly to their home cage at the end of the experimental session.

At the conclusion of initial period of training, the animals were implanted with cephalic recording chambers (Gray Matter Research, Bozeman, MT, USA) and a custom built 10-channel epidural ECoG array using sterile surgical technique [43, 14]. The ECoG array consisted of 4 mm Ag-AgCl electrodes (BioPac Systems Inc., Goleta, CA) fixed to 2×6 mm titanium bone screws that penetrated the skull to collect epidural and in some cases subdural signals from the overlying cortex, figure 2. The ECoG signals were collected from left and right occipital (LO and RO), temporal (LT and RT), frontal cortices (LFL, LFM, RFL and RFM) and midline structures (FZ, CZ). All neurophysiological signals were recorded using an RZ2 data acquisition system (Tucker Davis Technologies, Alachua, FL). The ECoG signals were recorded using low-impedance headstages (RA16LI and RA16PA) and Medusa preamplifiers at 1017.6 Hz and 508.6 Hz sampling rates and down-sampled to 508.6 Hz for the subsequent data analysis.

### 2.2. Vigilance task and experimental sessions

**Figure 1.**
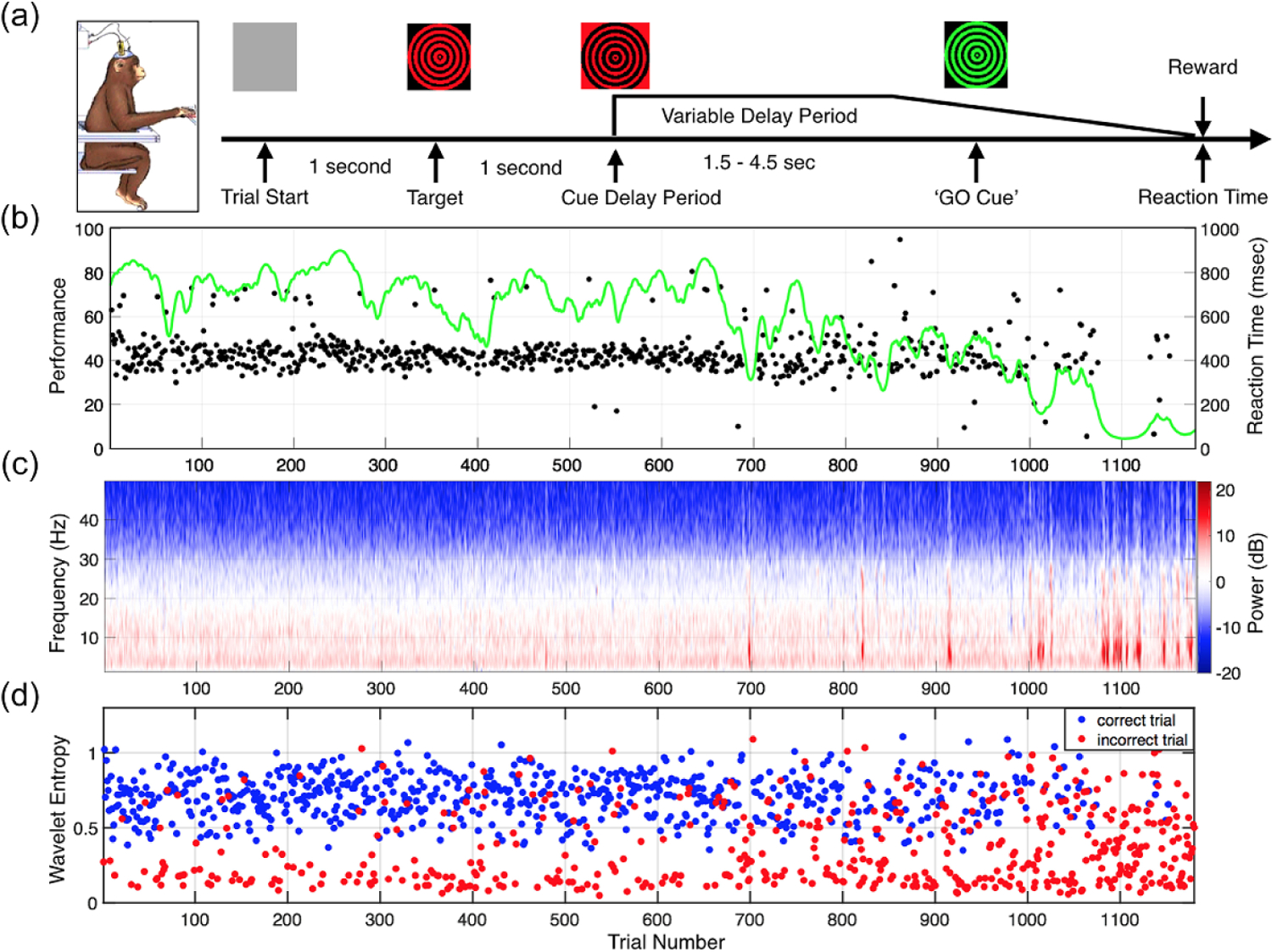
(a) Structure of the vigilance task. (b) Behavioral performance of NHP2 during 1180 trials. The performance estimate is shown as a smoothly varying green line and reaction times of correctly performed trials are plotted in black (total time on task: 128 min). (c) The power spectra of the signal collected from the midline frontal ECoG electrode (FZ) across trials. (d) The corresponding wavelet entropy calculated during the delay period for correct (blue) and incorrect (red) trials.

Two NHPs were trained to perform a visuomotor reaction-time task (i.e. vigilance task), that required sustained attention and fixation of a visual target over delay periods several seconds in duration. The vigilance task started with the appearance of the target (a 2 degree black/red checkerboard or dartboard) at one of 9 locations, chosen at random on each trial, on a CRT monitor positioned 113 cm in front of the animal. After a 1 second period of stable fixation, the target underwent color contrast reversal at 10 Hz for a variable delay period until switching to a black/green checkerboard or dartboard, as illustrated in figure 1(a). The transition to black/green from black/red was the ‘GO’ signal for the animal to make contact with an infrared touch switch (Crist Instruments) located within the primate chair, for a juice reward (0.2–0.4 ml). The variable delay period was randomly drawn from a normal distribution with mean of 2500 ms and standard deviation of 250 ms. A trial was considered to be incorrect if the NHP broke fixation prior to the ‘GO’ cue or touched the IR switch before or within 200 ms after the ‘GO’ cue or failed to respond within 800 ms after the ‘GO’ cue. ECoG signals were collected as the animals performed the vigilance task. All sessions in this study included periods of continuous central thalamic deep-brain stimulation (CT-DBS) [14], but here we restrict our analysis to trials during the non-DBS periods. In total, 99 experimental sessions were analyzed (55 for NHP1 and 44 for NHP2). DBS was not used during the experimental session shown in figure 1.

Performance of the vigilance task was generally high at the start of each recording session and gradually decreased over time, as shown in figure 1(b). Performance decrements typically included both an increase in the number of incorrect and/or incomplete trials and greater variance in reaction times. The power spectrum across each single trial during the first second of the delay period is shown in figure 1(c), while the corresponding wavelet entropy (as one of the proposed biomarkers, section 2.3.2), is shown in figure 1(d), indicating that the incorrect trials generally have lower values of wavelet entropy as compared with correct trials.

### 2.3. Methods and performance evaluation

We examined several time periods during each trial in a session to determine if the features that contribute to classification are influenced by the various stages of behavior performed on each trial, and how those changes influence classification. As shown in figure 1(a), we defined the following three periods of time for performance evaluation: 1. Pre-target period (from trial start to the appearance of the target); 2. Target period (1 s starting from the onset of the visual target); 3. Delay period (1 s starting from the onset of the cue for the start of the delay period). Our main results in this study are focused on the delay period.

#### 2.3.1. Eye movement signals

ECoG recordings of neural activity from alert subjects are impacted by eye movements, both by the electrical potential produced by the moving eye and any neurogenic activity generated by visuomotor processing [44, 45]. In this study, we applied independent component analysis (ICA) to reduce the eye movement contribution to the analyzed signals. Fast ICA was used to decompose the raw signal into independent components [46] and those that were determined to be correlated strongly with eye movements were removed (i.e. correlate with frontal channels). For this, we calculated the Pearson correlation between the components and the frontal channels LFL and RFL; if the correlation surpassed 0.1 (set empirically), the corresponding components were removed and the remaining components were transformed back to the original signal space (supplementary figure 1 (available online at stacks.iop.org/JNE/18/036001/mmedia)). We compared the characteristics and classification performance of ICA-corrected ECoG signals against those without the ICA pre-processing stage.

#### 2.3.2. Neural biomarkers of fatigue

In order to accurately and robustly predict the animals’ performance in the vigilance task, we extracted the following set of biomarkers (table 1) from each ECoG channel in the examined epoch of the trials: Spectral power in multiple frequency bands as detailed in table 1; Wavelet entropy, which reflects the degree of order/disorder associated with a multi-frequency signal [47] and has been shown to differentiate between different brain states [48, 11]; the Hjorth parameters indicating the statistical properties of neural signal in the time domain [49], including the Hjorth activity as a measure of signal variance, Hjorth mobility representing the mean frequency of a signal, and Hjorth complexity representing the frequency changes over time; Phase-amplitude coupling (PAC) between the phase of theta (here, 3–7 Hz) and amplitude of gamma (here, 70–130 Hz) band activity [50–52]. We also extracted the instantaneous amplitude (IA) over the delta band by computing the modulus of the analytic signal across the delta frequency range, as well as the instantaneous frequency (IF) indicating the shift in the frequency content in theta-alpha band (here, 4–14 Hz [53]), and the ratio between these two values. The instantaneous frequency was calculated by taking the time derivative of the phase of the analytic signal. The IA/IF ratio has been shown to be effective in pinpointing the onset of drowsiness in ECoG studies on epilepsy patients [53]. All of these biomarkers from individual ECoG channels were included as features for classification.

In addition to single channel measures, we also included as features for the classification stage spectrotemporal connectivity measures involving multiple ECoG channels. We extracted the partial directed coherence (PDC) within the delta, theta, alpha and beta bands [23] across all pairs of channels, and the global coherence [54] within all sub-bands by utilizing all of the ECoG channels. We also computed the Phase Locking Index (PLI) [55] between the pairs of ECoG channels within delta, theta, alpha and beta bands.

#### 2.3.3. Feature selection and classification

In this work, we used the gradient-boosting decision tree ensemble (the XGBoost package in Python) as the classifier [56], given its high performance in several prior studies on neurophysiological signals [11, 57–60]. Particularly, this model was shown to perform best among other ML models in epileptic seizure detection from ECoG [57, 61] and Parkinsonian tremor detection from LFP [11, 59]. Moreover, in our preliminary study on this ECoG dataset, XGB outperformed several other ML models such as linear discriminant analysis (LDA) and support vector machine (SVM) with different kernels. We built a gradient-boosting model with 30 trees, and a maximum depth of 4 to avoid overfitting, and trained a subject-specific model to predict the trial outcome. The number and maximum depth of the trees were fixed based on our initial study on this dataset that led to a high classification performance in both animals. We used the F1 score to report the performance of the classifier in detecting incorrect trials, defined as the harmonic mean of sensitivity (TP/(TP+FN)) and precision (TP/(TP+FP)), i.e. $\text{F1}=2\cdot \left(\frac{\text{precision}\cdot \text{sensitivity}}{\text{precision}+\text{sensitivity}}\right)$, with TP representing the number of true positives, FP representing the number of false positives, and FN representing the number of false negatives. Here, the target positive class is the incorrect trial. The F1 score ranges from 0 to 1, with higher values representing a better performance. A nested 5-fold cross-validation method was used to calculate the F1 score, in which the inner loop was used to tune the parameters of each model (e.g. the optimal number of features as described below), and the outer loop to estimate the performance on the test set. We also report the accuracy, sensitivity, and specificity of our classifier, all measured with 5-fold cross validation.

While the inherent feature selection capability of the tree-based gradient boosting algorithm allowed a broad feature search in our study, a more rigorous feature selection step could further improve the performance and reduce the risk of overfitting. Here, a wrapper-based approach [62] was used to identify the most discriminative features as they were fed to the XGB model. The algorithm starts by finding the best single feature that achieves the highest F1 score on the training set, measured by 5-fold cross-validation (the inner loop of the nested cross-validation). It then continues to add the next ‘best feature’ in each iteration, until a near-optimal performance is achieved. The final performance is then reported on the held-out test set using the selected features.

#### 2.3.4. Statistical analysis

One-way ANOVA with repeated measures was used to study the difference in classification performance obtained by different features. We used the MATLAB function ANOVA (MATLAB version R2018b) for the statistical analysis performed in this work. For cases with a significant main effect, Bonferroni correction was used for post-hoc analysis. A paired sample t-test was used to infer whether the performance of our proposed HOST feature set was significantly higher than that of using only the spectral power features.

## 3. Results

**Figure 2.**
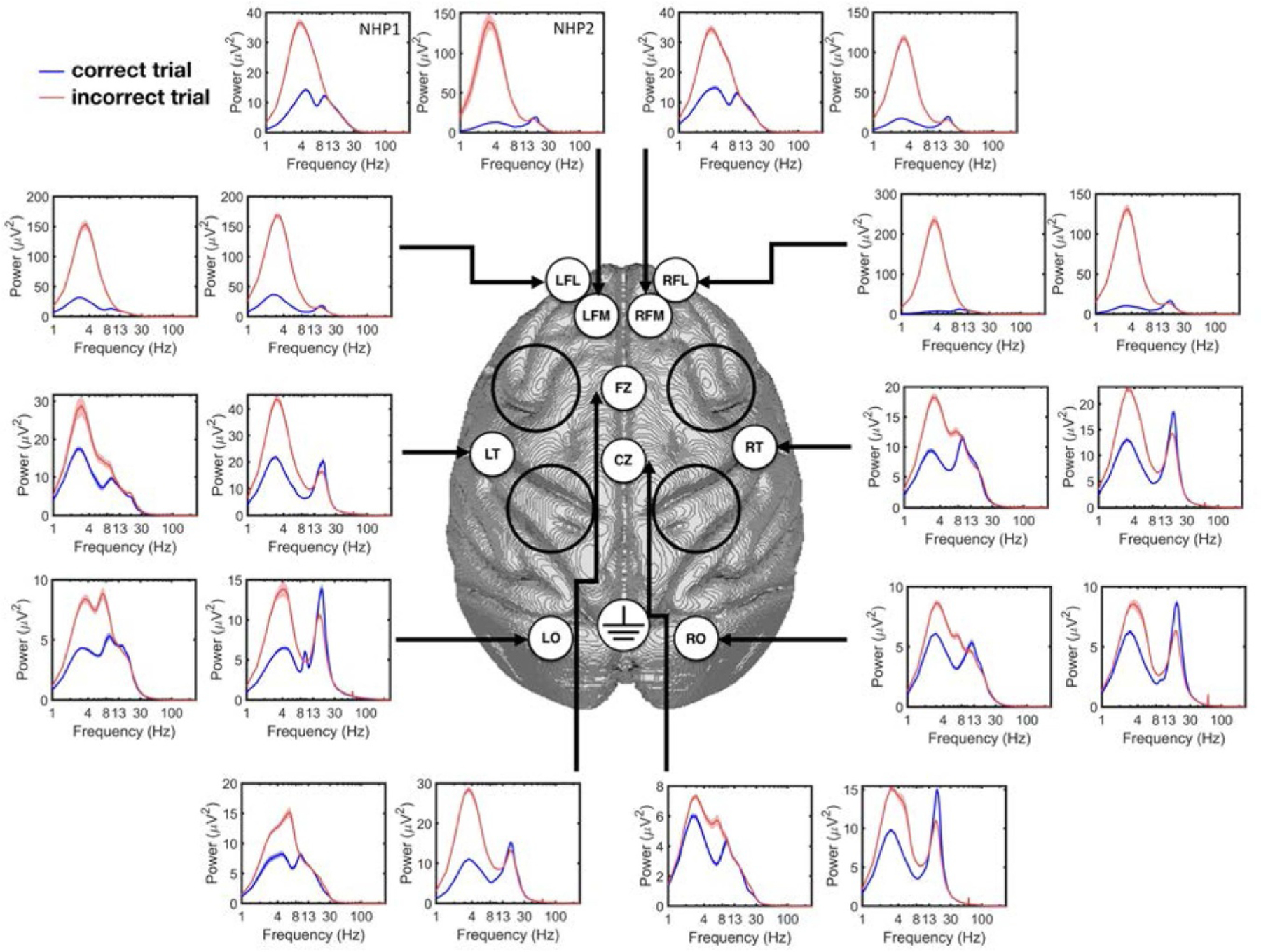
The power spectrum of correct and incorrect response trials from each ECoG channel for NHP1 and NHP2. In each subplot, the left figure corresponds to NHP1 while the right one corresponds to NPH2. Note that the range of *y*-axis is different between the two NHPs and on different electrodes. The black circles illustrate the locations of the cephalic chambers implanted in both animals.

**Figure 3.**
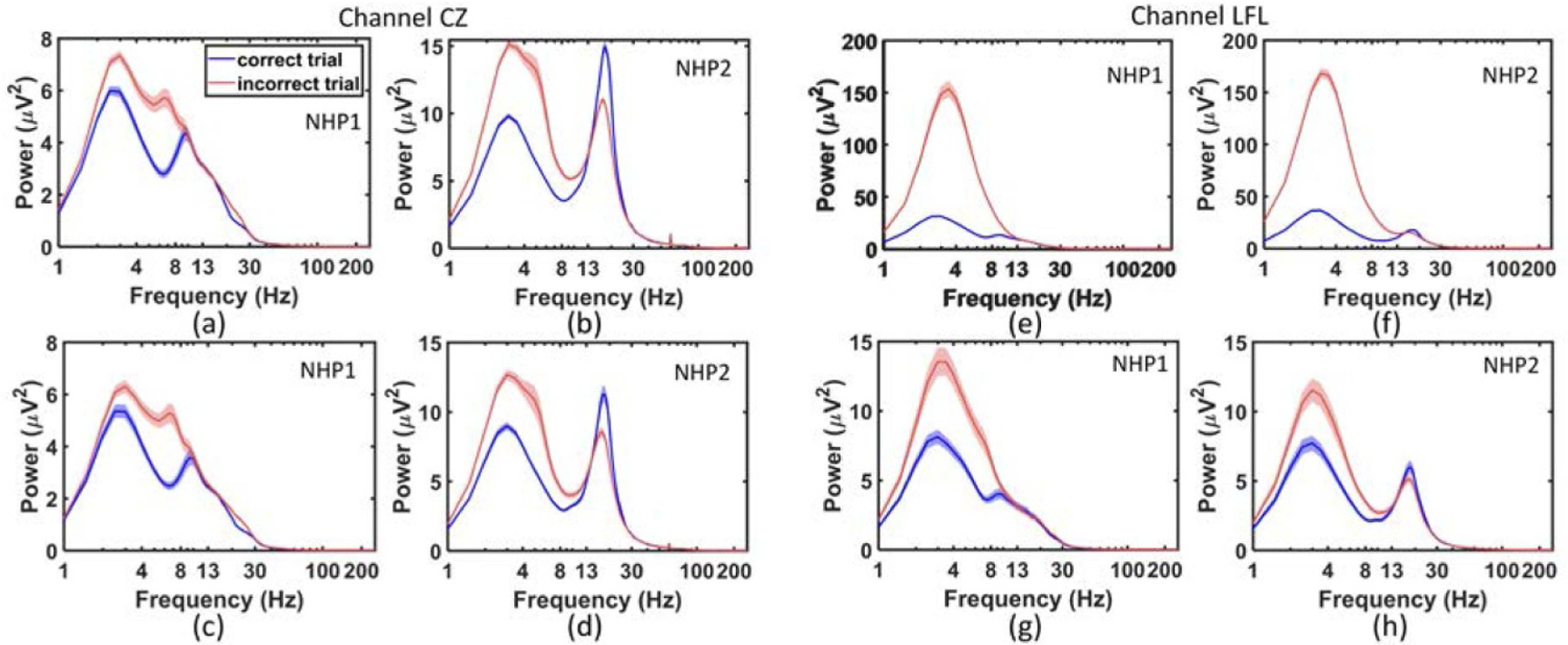
The power spectrum in NHP1 and NHP2 from midline ECoG channel CZ (left) and the frontal ECoG channel LFL (right). (a), (b) The power spectrum at CZ in NHP1 and NHP2, without using ICA. The thickness of the curves for the spectra indicates the standard error of the power estimates across sessions. (c), (d) The power spectrum at CZ in NHP1 and NHP2 with ICA. (e), (f) The power spectrum at LFL in NHP1 and NHP2 without using ICA. (g), (h) The power spectrum at LFL in NHP1 and NHP2 with ICA. Note that the range of *y*-axis is different before and after ICA.

The power spectrum correct and incorrect trial responses for each recording channel are shown in figure 2, illustrating higher spectral power (below 13 Hz) in incorrect trials as compared to the correct trials (the time-frequency plot of each channel during the delay period for correct and incorrect trials is shown in supplementary figure 2). The use of ICA does impact the distribution of power in the spectra calculated for ECoG signals in specific channels. Figure 3 shows the power spectrum for correct and incorrect trials in NHP1 and NHP2, at channel CZ without using ICA (figures 3(a) and (b)), and with using ICA (figures 3(c) and (d)). It can be seen that in both NHPs, the power spectrum for incorrect trials is significantly higher than for correct trials, between 1 and 10 Hz. While the ICA step did not significantly change the power spectrum at channel CZ, the power spectrum for frontal channel LFL is markedly reduced with the application of ICA, figures 3(e)–(h). Note however that the power spectra for incorrect trials is still significantly higher than that for the correct trials, in the lower frequency bands (<10 Hz).

**Figure 4.**
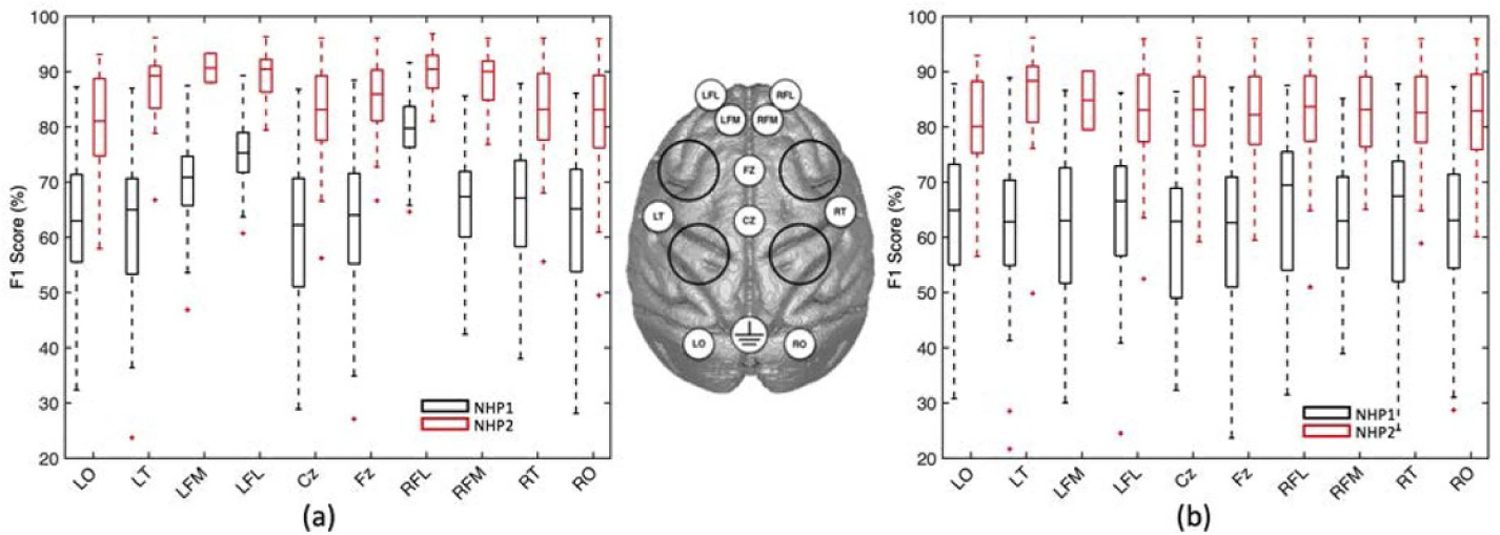
Channel importance analysis. (a) The performance of the classifier for NHP1 and NHP2, using all features from single electrodes. The error bars indicate the standard error. (b) The performance of a classifier built on individual channels for NHP1 and NHP2 after applying ICA. The use of ICA stabilizes the contribution of ECoG channels to the performance of the classifier. Note that without ICA, the LFL and RFL channels located at the front of the head nearest the eyes, make a significant contribution to classifier performance in both animals.

The performance of the classifier when only one channel is used for classification, is shown in figure 4. Without ICA, the frontal channels such as LFL and RFL exhibited a higher performance compared with other channels, figure 4(a). Following ICA removal, however, all channels obtained a nearly similar performance, as shown in figure 4(b).

**Figure 5.**
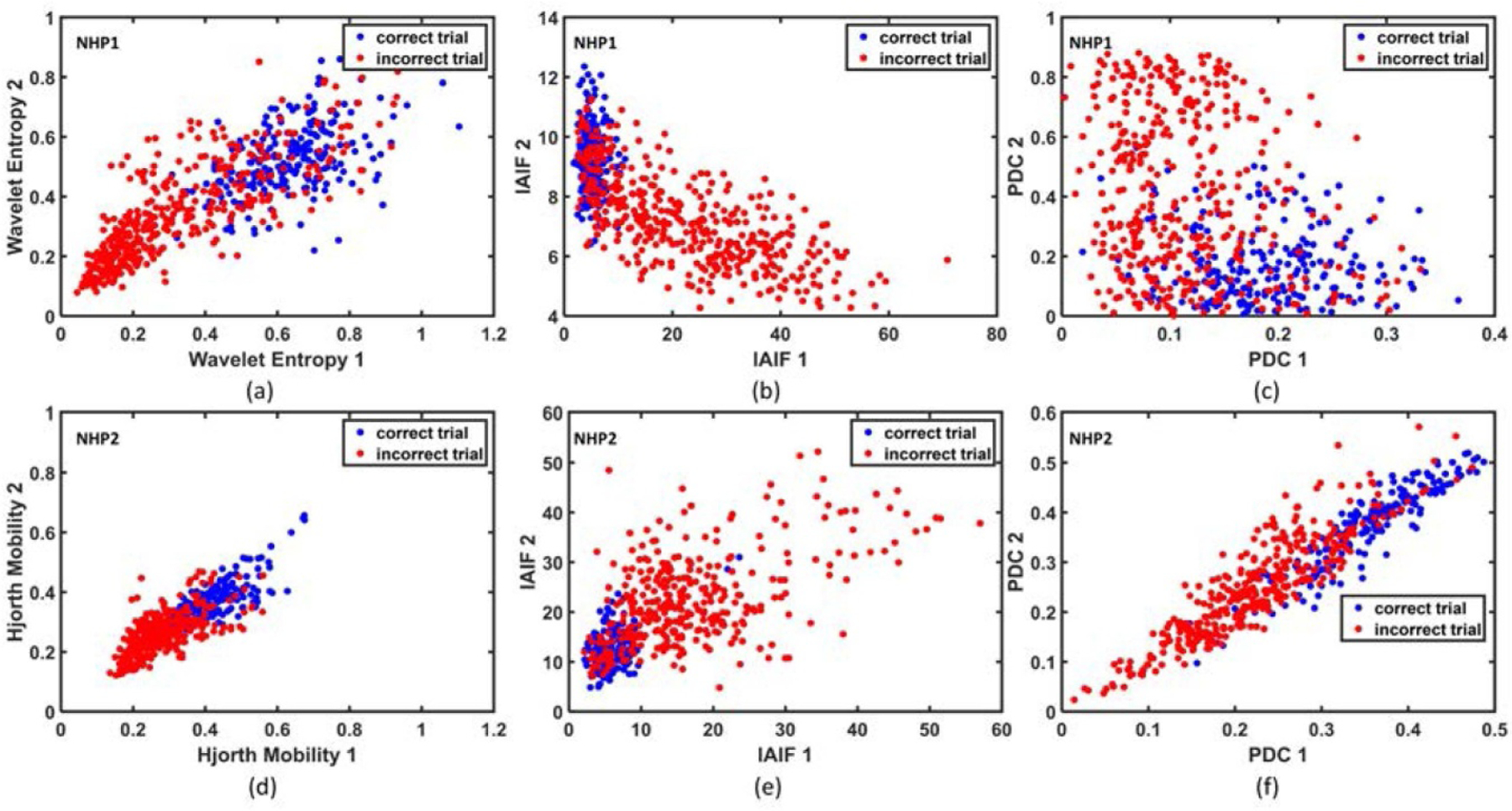
Feature distribution between correct and incorrect trials showing the separability of two classes. (a) Distribution of wavelet entropy features in NHP1. (b) Distribution of IAIF features in NHP1. (c) Distribution of PDC features in NHP1. (d) Distribution of Hjorth mobility features in NHP2. (e) Distribution of IAIF features in NHP2. (f) Distribution of PDC features in NHP2. In each case, the two features that exhibit the highest *R*^2^ value with class label are used to plot the feature distribution.

The feature distribution of several biomarkers from table 1 is shown in figure 5, for the two NHPs. The two features with the highest *R*^2^ value are shown in each case, where *R*^2^ represents the square of Pearson correlation coefficient between a feature and the corresponding label (i.e. correct or incorrect class). As illustrated in this figure, the wavelet entropy features (wavelet entropy 1 and 2, extracted from two different channels) were highly discriminative for correct versus incorrect trials of NHP1, with the incorrect trials exhibiting a lower wavelet entropy, figure 5(a). Similarly, the Hjorth mobility is a highly discriminative feature for NHP2, where incorrect trials have a lower mobility compared to correct trials, figure 5(d).

**Figure 6.**
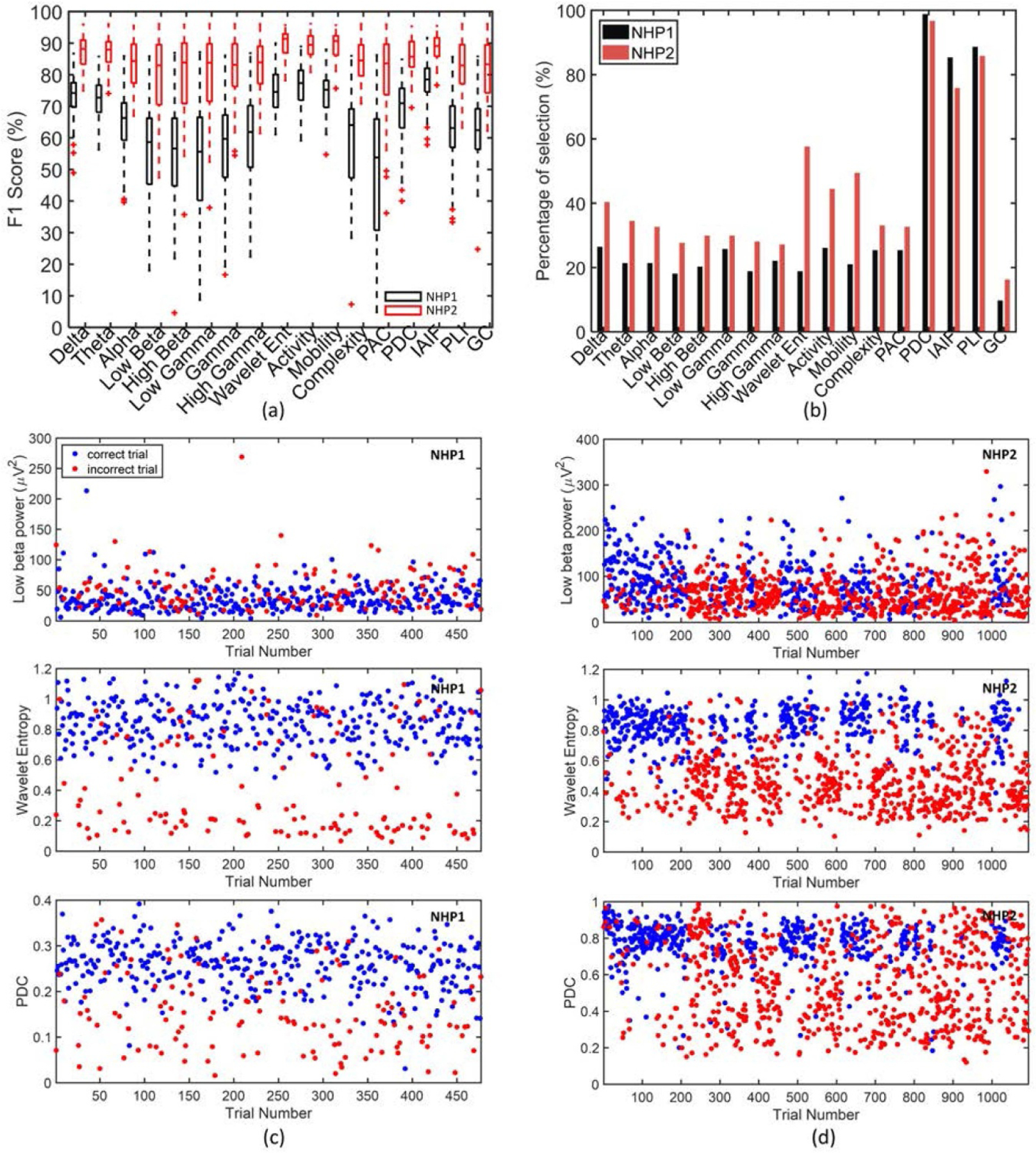
(a) The boxplot of individual feature performance, using all electrodes in NHP1 (black) and NHP2 (red). (b) The percentage of feature selection by wrapper method. If a feature was selected more than once in a session (e.g. from two different channels), it was counted as one selection. (c) The feature dynamics across trials for one session from NHP1, with the upper of the three plots corresponding to low beta power, the middle of the three plots showing the wavelet entropy, and the lower plot showing the PDC. (d) The feature changes across trials for one session from NHP2, with the upper plot corresponding to low beta power, the middle plot showing the wavelet entropy, and the bottom plot showing the PDC. For both (c) and (d), correct trials are blue and incorrect trials are red.

In contrast to methods such as deep neural nets (DNNs), decision trees are generally interpretable and provide useful insights on the contribution of different features to the classifier’s performance. In this study, we first compared the performance of individual features in classification, each extracted from all channels or from a combination of channels. We observed a significant difference between the performance of various biomarkers in NHP1 (the black boxplots in figure 6(a), *F*(16,864) = 71.0, *p* = 4.1*e*−145), and NHP2 (the red boxplots in figure 6(a), *F*(16,688) = 27.0, *p* = 2.8*e*−62). The IAIF feature obtained an average classification performance of 77.6%±7.6% in NHP1, while the wavelet entropy obtained an average performance of 90.0%±4.2% in NHP2, outperforming other features in each case. Furthermore, figure 6(b) shows the percentage of selection by wrapper method for each feature type in table 1 (i.e. in what percentage of sessions a feature is selected by the wrapper algorithm). This figure shows that PDC and PLI were frequently selected as predictive biomarkers in both NHPs. figures 6(c) and (d) show the feature dynamics across trials for both NHPs. Interestingly, we can see that compared with a widely used feature such as low beta power, the features of PDC and wavelet entropy exhibited a higher discriminative power between correct and incorrect trials in this study.

**Figure 7.**
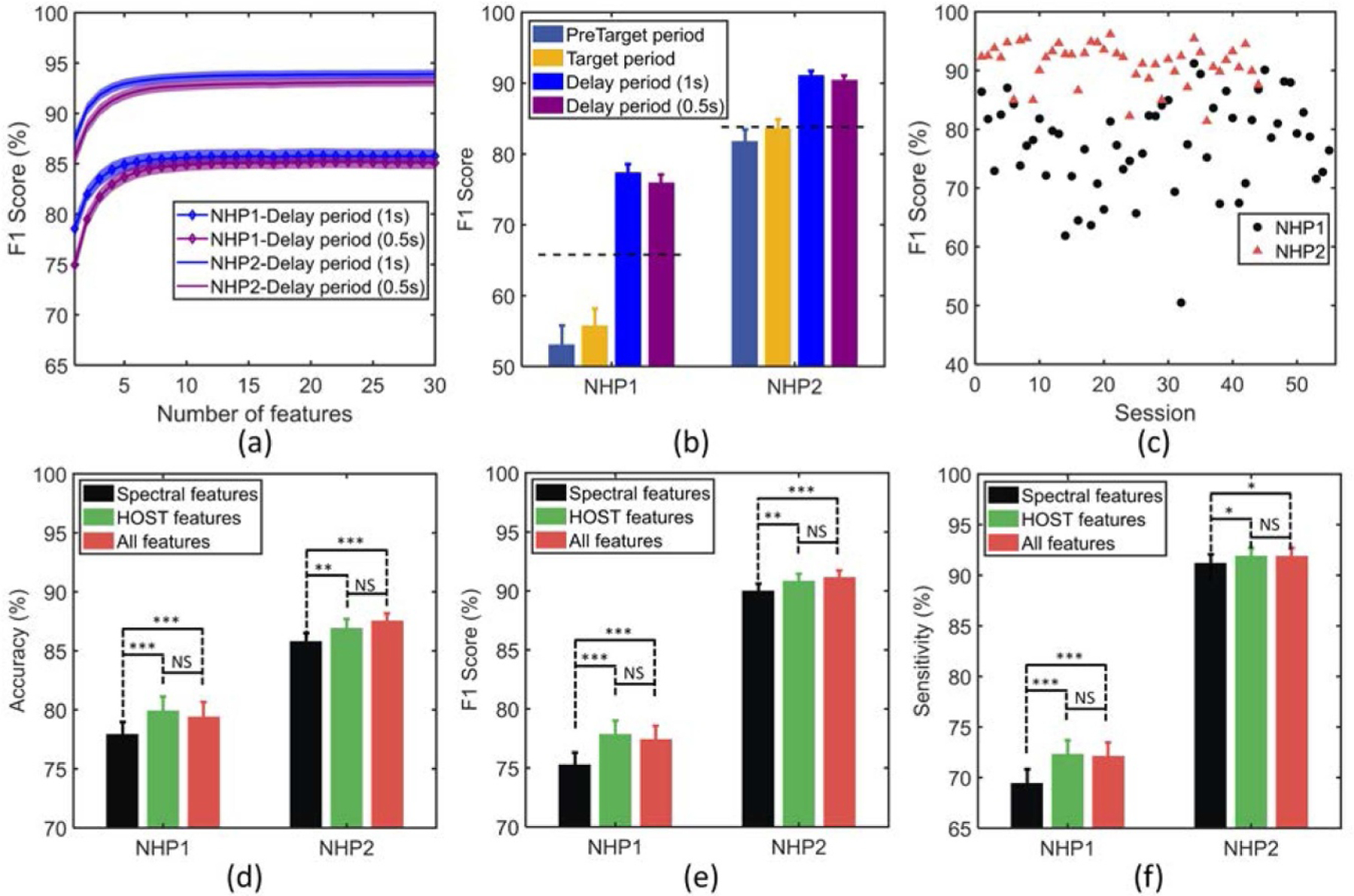
(a) Training performance of the classifier during delay periods versus number of features. The most predictive features are iteratively added to the classifier using wrapper approach (the shaded area indicates the standard error over sessions). (b) Test performance of the classifier in various time periods for NHP1 and NHP2. The horizontal dashed lines indicate the baseline performance obtained by an all-positive detector for comparison (i.e. when all trials are detected as incorrect). (c) Classifier performance across sessions for NHP1 and NHP2. (d) Comparison of classification performance for spectral versus HOST features, and the combination of both feature sets. The performance was measured by metrics of accuracy (d), F1 score (e), and sensitivity (f), in both NHPs. The top performing features selected by wrapper method were used to plot (d)–(f). Note: *** *p* < 0.001, ** *p* < 0.01, * *p* < 0.05, NS: non-significant.

Figure 7(a) shows the performance of the classifier during delay period (using the first 1 s or 0.5 s of delay period), as the number of features selected by wrapper method increases (verified on the training data). In both NHPs, the cross-validation performance saturates by using as few as 5–10 features per session. Moreover, a window size of half a second during delay period showed comparable performance to a one second window in the same period. The selected features were next used to measure the performance on the held-out test set for each NHP (figure 7(b)), using the activity from various time periods prior to the ‘GO’ response. As shown in figure 7(b), in both NHPs the classifier performance over delay period is significantly higher than the performance over target and pre-target periods, surpassing the baseline performance obtained by an all-positive detector (the dashed line in figure 7(b), corresponding to a baseline F1 score of 66% and 84 % for NHP1 and NHP2, respectively). figure 7(c) shows the performance of the classifier over delay period (1 s) and across sessions, reaching an average F1 score of 77.5%±8.2% for NHP1 and 91.2%±3.6 % for NHP2, respectively. Moreover, we compared the classification performance using conventional band power features (i.e. including the spectral power features from multiple frequency bands as detailed in table 1), with that of using higher order spectral-temporal (HOST) features (all features in table 1 excluding the spectral power features), and with the combination of both feature sets. As depicted in figures 7(d)–(f), for NHP1, the classification accuracy, F1 score, and sensitivity of the HOST feature set were all significantly higher than those of the conventional spectral features (*p* < 0.001); forNHP2, the classification performance of the HOST features was significantly higher than that of spectral features (*p* < 0.001 for accuracy, *p* < 0.01 for F1 score, and *p* < 0.05 for sensitivity). Furthermore, adding the spectral features to the HOST feature set did not significantly improve the classification performance.

The behavioral performance at the start of the experiments was 65.5%±14.4% and 66.0%±19.4 % for NHP1 and NHP2, respectively (the number of correct trials divided by the total number of trials at the beginning of each session prior to the first use of DBS, mean ± SD). Our proposed feature set and machine learning approach obtained an F1 score of 77.5%±8.2% for NHP1 and 91.2%±3.6% for NHP2, in detecting incorrect response trials. Furthermore, table 2 summarizes the classification performance over the delay period (1 s), using different metrics such as sensitivity, specificity, accuracy, and balanced accuracy (the mean of sensitivity and specificity, to account for the unbalanced distribution of correct and incorrect trials across subjects).

## 4. Discussion

In this study, we developed a classifier for predicting correct vs incorrect trial outcomes based on a set of biomarkers extracted from multi-channel ECoG recordings of brain activity. By employing a subset of 5–10 optimal features, trial outcomes were predicted with high accuracy, using the spectrotemporal activity recorded across the brain during the early part of the delay period in the task. Power in the lower frequency bands (delta and theta) contributed to classification in both NHPs. We considered that eye movement potentials may significantly contribute to the overall power in the delta band. So we used ICA to remove the eye movement potentials (supplementary figure S1), and as expected, the contribution of the delta band was significantly reduced. This finding raises a general point about the analysis of ‘noisy’ ECoG and EEG data. Multi-channel brain signals may be contaminated by behavioral movement signals, both as artifacts and movement related neurogenic potentials. While these noise components may not tell us directly about brain states of sustained attention or mental effort, they may be useful for identifying behavioral states of distraction and inattentiveness that are often associated with intrusive eye movements.

We acknowledge that the use of eye movements as measure of attentional state must be qualified with a specification of the nature of the behavioral task at hand. For example, tasks requiring fixation of a target that will provide a go-signal or instructions for the correct behavioral response in a trial, recruit a number of cortical and subcortical brain areas to produce the control signals that suppress spontaneous eye movements, or saccades to irrelevant features or the wrong visual target [63]. Inactivation of the superior colliculus [64] or the basal ganglia [65] with muscimol (a chemical agent that inhibits the activity of neurons) will increase the frequency of intrusive saccades and the inability to suppress a saccade to a distracting visual signal. We suggest that mental fatigue acts much like an increase in inhibition of the brain areas involved in suppressing saccades, but through a reciprocal process; i.e. a withdrawal of neural excitation in these areas, not an increase in inhibition. However, if a task requires that the subject search a visual scene for a target with their eyes, then attentiveness would typically be positively correlated with an increase in the number of saccadic eye movements [66]. On the other hand, as a subject becomes drowsy, eye movements can decrease to the point where eye movements cease altogether, a phenomenon known as a blank stare [67]. In the task used in the experiments described here, the NHPs were required to restrict their eye movements to within a few degrees of a fixation target in order to perform the task correctly. Thus, eye movements during the delay period could be classified as intrusive saccades and their appearance correlated closely with poor task outcome.

Several human EEG-based studies have used frontal theta and midline alpha power as features for detecting changes in brain state associated with fatigue during driving and other experimental tasks [17, 18, 24]. Real-time monitoring of these signals was successfully integrated into real-time detection and reporting systems. In this study, we employed a variable delay period reaction-time paradigm to induce mental fatigue and influence task performance, and introduced new biomarkers for quantifying correct and incorrect responses during the task. In addition to relatively simple spectral features such as theta power, we found that other complex spectrotemporal features also contributed significantly to the performance of the classifier. For example, among single channel features, measures of the wavelet entropy of the spectrogram were important. Multi-channel measures computed across the spectrum, such as global coherence, also aided in classification. However, the wavelet entropy, Hjorth activity and mobility, and IAIF features were best at differentiating correct and incorrect responses in the two NHPs. Moreover, the PDC, PLI, IAIF and wavelet entropy were among the features most often selected during the feature selection process. Interestingly, the cross-regional connectivity measures such as PDC and PLI were frequently selected as predictive biomarkers of performance in both NHPs (figure 6(b)). This is consistent with recent studies that employ cross-regional coherence for decoding cognitive control tasks, where single-region spectral features only obtain a chance level of performance [31]. There is increasing evidence that network measures play a key role in predicting mental state and task engagement [68], and may provide a robust marker to control adaptive stimulation therapies for mental [31, 69] and movement disorders [70].

Our long-term goal is to robustly detect shifts in brain state, specifically to identify periods of mental fatigue in order to intervene using deep brain stimulation to shift global cortical activity into a state where cognitive resources can be better recruited. We show here that a set of spectrotemporal biomarkers can be used by a classifier to predict trial outcome, and that delay period activity, as opposed to earlier periods in the trials, provides the best predictive samples of brain activity (figures 7(a) and (b)), and that classifier performance can fluctuate across experimental sessions (figure 7(c)). Our multi-channel ECoG spectrotemporal biomarkers were sensitive to the eye movement potentials generated during the task, and as a consequence, the classifier could exploit this sensitivity to reach high predictive accuracy for trial outcome. The eye movement potentials were most prominent in the four frontal electrodes (LFL, RFL, LFM, RFM). The signals in these channels contributed significantly to the classifier’s performance. If we removed the four frontal channels, and relied only on the remaining six (as done in our preliminary study [71]), classifier performance (F1 score) was reduced to 63.8 % and 87.2% for NHP1 and NHP2, respectively. The sensitivity of the classifier for the two NHPs when frontal channels were removed is 63.2% and 90.6 %, respectively, while the specificity values are 64.0% and 45.3%, respectively. It is important to note that the difference in the obtained levels of accuracy, F1 score, sensitivity and specificity for the two NHPs is partially due to the unbalanced distribution of correct and incorrect trials in NHP2 (202±129/518±165) while the distribution of trials in NHP1 is more balanced (254±131/270±148). As a result of higher number of incorrect trials in NHP2, we observe a higher sensitivity (TP/(TP±FN)) and a lower specificity (TN/(TN±FP)), as well as a higher F1 score and accuracy. Thus, the ‘balanced accuracy’ measure (the mean of sensitivity and specificity) was also reported which is a common approach to evaluate the performance on unbalanced datasets. There was around 20% difference in predictive accuracy between NHP1 and NHP2, however they have similar balanced accuracy (77.0% vs. 79.3%) and the predictive accuracy of both NHPs was significantly above random level.

The performance of the single-trial classification can be influenced by different periods of the task period. The pre-target period (Trial Start in figure 1) and the target period (Target in figure 1) showed a similar performance of around 54% in NHP1 and 83% in NHP2. Furthermore, the performance during the delay period was around 20% higher than the target period in NHP1 and around 8% higher in NHP2. By employing a short time window of half a second, we found that we can achieve similar performance as compared with a window of one second in duration in both NHPs. A shorter window would have a faster detection and lower latency in real-time settings, and should be considered for future closed-loop systems using these candidate biomarkers.

We limited our current work to single-trial classification in an offline setting, and focused on the delay period of a simple reaction time task paradigm. In the current work we sought to answer if the correct and incorrect behavioral responses could be correctly predicted from neural activity in a time period occurring several seconds prior to the actual motor response. By integrating the conventional spectral features and several new features, we were able to correctly predict task performance by classifying the brain states associated with correct and incorrect behavioral responses. These results provide strong support for developing a system that can use multi-channel ECoG data to identify brain states associated with poor attention and fatigue.

The focus of current study is on high-accuracy subject-specific prediction in each NHP, where we observe different levels of predictive performance in the two animals. In order to generalize the proposed approach across subjects, one may train the classifier on a sufficiently large number of NHPs and test the resulting model on a new subset of animals, an approach that is being investigated in our future work.

## 5. Conclusion

In this study, we employed modern machine learning techniques to analyze ECoG signals sampled diffusely across the cortical surface of two NHPs as they performed a vigilance task requiring sustained attention over extended periods of time. We identified several features in the ECoG signals that robustly predicted behavioral performance in both animals and across recording sessions. Specifically, we found that higher-order spectral-temporal (HOST) features outperformed conventional spectral features. These results support our efforts of developing robust algorithms to predict performance and periods of mental fatigue in behaving animals. In principle, the approach developed here could be used in adaptive therapeutic interventions, like DBS, to restore arousal regulation, which is impaired in patients with structural brain injuries. However, the translation of this approach to humans will require studies that focus on the development of biomarkers linked to impaired cognition and executive function in patients.

## Supplementary Material

Supp Fig 1

Supp Fig 2

## Figures and Tables

**Table 1.** Neural biomarkers extracted from ECoG.

Feature	Description
1. Delta	Band power in (1–4 Hz)
2. Theta	Band power in (4–8 Hz)
3. Alpha	Band power in (8–13 Hz)
4. Low Beta	Band power in (13–20 Hz)
5. High Beta	Band power in (20–30 Hz)
6. Low Gamma	Band power in (30–45 Hz)
7. Gamma	Band power in (60–90 Hz)
8. High Gamma	Band power in (100–200 Hz)
9. Wavelet Entropy	$E=-\sum_{i=1}^{n}p_{i}\ln\left(p_{i}\right)$, where *p*_*i*_ is the relative wavelet energy, and *n* is the decomposition level (*n*=4, and the ‘db4’ wavelet is used for the decomposition)
10. Hjorth Activity	*var*(*y*(*t*)), where *y*(*t*) is the input signal
11. Hjorth Mobility	$\sqrt{var\left(\frac{dy\left(t\right)}{dt}\right)/var\left(y\left(t\right)\right)}$
12. Hjorth Complexity	$\text{mobility}\left(\frac{dy\left(t\right)}{dt}\right)/\text{mobility}\left(y\left(t\right)\right)$
13. PAC	$\frac{\left[\log\left(N\right)-H\left(P\right)\right]}{\log\left(N\right)}$, where *N* is the number of bins dividing the phase, *H*(*P*) is the Shannon entropy of the amplitude distribution, and *P* is the amplitude distribution
14. PDC	$pdc_{ij}=B_{ij}\left(f\right)/\sqrt{b_{j}^{*}\left(f\right)b_{j}\left(f\right)}$, where *B* is the Fourier transform of multivariate autoregressive (MVAR) model coefficients, *b*_*j*_ is the *j*th column of *B*, and * represents the transpose and complex conjugate operation (1≤*i*, *j*≤10 for 10 ECoG channels, and *i*≠*j*
15. IAIF	Instantaneous amplitude (IA) over delta band, instantaneous frequency (IF) over theta-alpha band, and the ratio of IA and IF (i.e. three features per ECoG channel)
16. PLI	$\frac{1}{T}\sum_{t=1}^{T}e^{j\Delta \theta_{t}}$, where Δ*θ*_*t*_ is the phase difference between two signals at time *t*, and *T* is the total trial time
17. GC	Global coherence as the ratio of largest eigenvalue of the cross-spectral matrix to the sum of all eigenvalues

**Table 2.** Number of trials and classification performance for each NHP (mean ± SD).

Subject	#Correct/incorrect trials	Accuracy	Balanced accuracy	Sensitivity	Specificity
NHP1	254 ± 131/270 ± 148	79.5% ± 8.9%	77.0% ± 9.9%	72.2% ± 9.6%	82.0% ± 20.6%
NHP2	202 ± 129/518 ± 165	87.6% ± 3.9%	79.3% ± 11.6%	92.0% ± 5.2%	66.6% ± 25.7 %
